# Outcomes of Multi-disciplinary Management of Metastatic Renal Cell Carcinoma

**DOI:** 10.7759/cureus.5901

**Published:** 2019-10-13

**Authors:** Michael Yan, Richard Gregg, Aamer Mahmud

**Affiliations:** 1 Radiation Oncology, Kingston Health Sciences Centre, Kingston, CAN; 2 Oncology, Cancer Centre of Southeastern Ontario at Queen's University, Kingston, CAN; 3 Radiation Oncology, Cancer Centre of Southeastern Ontario at Queen's University, Kingston, CAN

**Keywords:** renal cell carcinoma, radiotherapy, multi-disciplinary, metastatic disease, palliative care

## Abstract

Metastatic renal cell carcinoma (mRCC) is associated with a poor prognosis. It is traditionally a treatment-resistant disease necessitating multi-modal treatment and close follow-up. We herein report a case of mRCC in a patient who was managed closely by a multi-disciplinary team and still retained a very good performance status and treatment response three years after diagnosis. We highlight the importance of close monitoring, switching systemic therapies at progression, early palliative radiotherapy, and patient education in controlling disease burden and maintaining quality of life in patients with mRCC.

## Introduction

Metastatic renal cell carcinoma (mRCC) has historically been associated with dismal prognosis, with five-year survival rates of 8% among patients with metastatic disease. About one-third of patients with RCC present with regional or distant metastases upfront [1]. Approximately 20-25% of patients treated radically recur with distant disease [2,3].

Traditionally a treatment-resistant disease, modern management of mRCC necessitates a multimodality approach. An arsenal of treatment techniques including surgery, radiotherapy, radiofrequency or cryoablation, chemotherapy, molecular therapy, and immunotherapy are used [1,4].

We present a case of metastatic renal cell carcinoma managed in a multidisciplinary setting involving urology, palliative care, as well as medical and radiation oncology, with multiple local and systemic treatments. We will also provide a review of the literature for current management paradigms for mRCC.

## Case presentation

**Figure 1:**
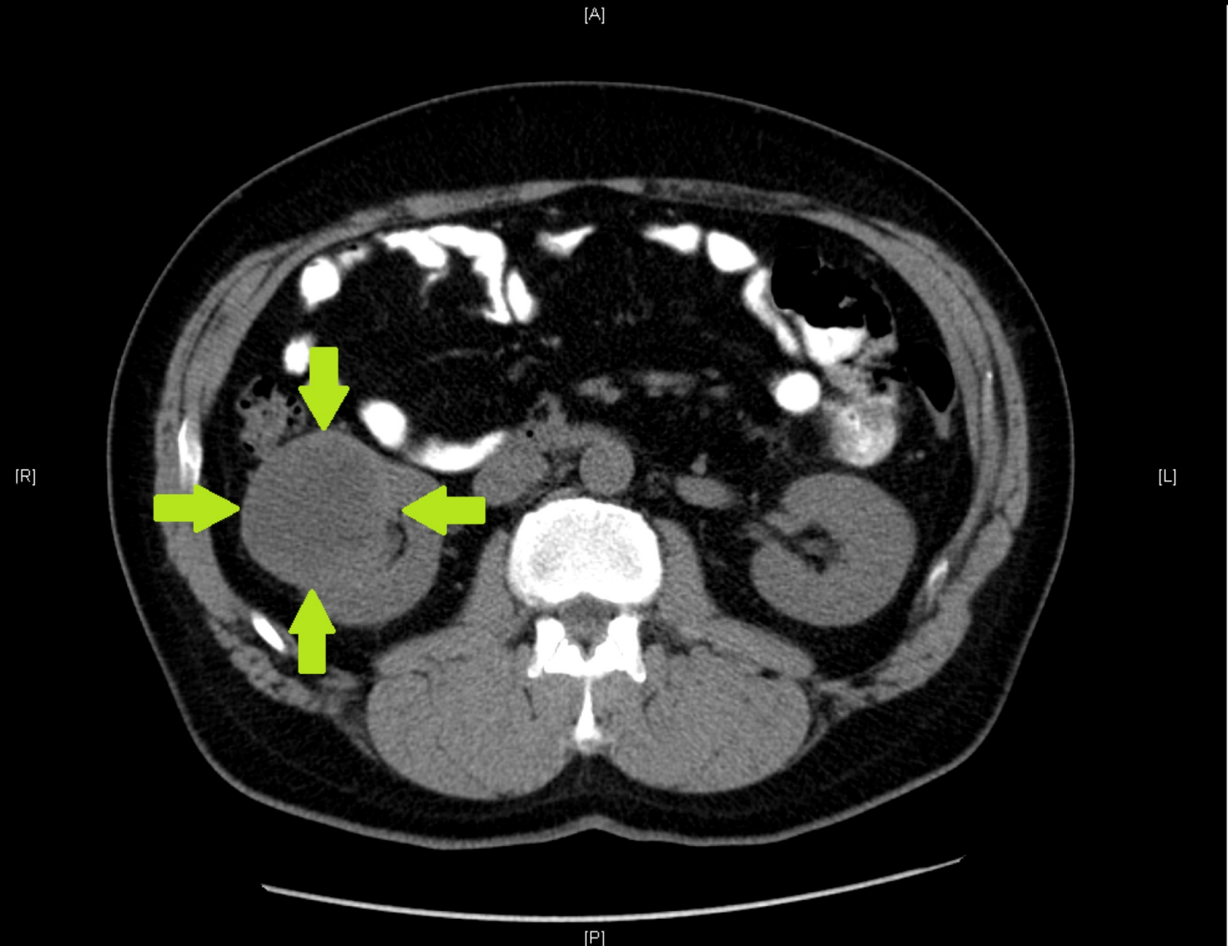
CT abdomen Axial CT image shows large renal cell carcinoma arising from the right kidney.

In September 2007, a previously healthy 48-year-old man presented with gross hematuria and right flank pain. He was found to have a 6.6 x 6.1 x 5.9 cm (CC x TR x AP) mass in the right kidney on CT imaging (Figure 1).

One week later, he underwent a laparoscopic nephrectomy, where a 5.5-cm renal cell carcinoma (RCC) was found on pathology. Histologically, it was a clear cell carcinoma with a Fuhrman grade of 3/4. Resection margins were widely negative.

In May 2015, he was admitted to hospital for diverticulitis. A CT scan performed incidentally showed multiple bilateral pulmonary nodules. These were biopsied, confirming recurrence of his RCC. No other sites of metastasis were observed.

He was referred to the regional cancer center and a decision was made not to initiate systemic therapy after discussion with the patient, who was completely asymptomatic at the time. Instead he was scheduled for monitoring.

In January 2016, surveillance imaging showed progression of the lung metastases and evidence of bone metastases. The patient also reported lower back pain consistent with his imaging findings. A bone scan showed an iliac lesion, as well as disease in his right rib, humeral head, distal femur, olecranon, and left ribs. He was initiated on Sunitinib. A referral was made to radiation oncology for a course of palliative radiotherapy to his symptomatic sites of bone metastasis.

The patient continued his follow-up in clinic with medical oncology and palliative care. He did develop a pathologic fracture of the right humerus requiring orthopedic intervention in August 2016. Subsequently, he had a spinal cord compression in November 2017 treated with laminectomy. In June 2018, he underwent excision of a metastasis in his right distal femur followed by total right knee arthroplasty.

His systemic therapy was switched from Sunitinib to Nivolumab after four cycles, then to Everolimus after seven cycles, and finally to Axitinib after nine cycles. He most recently received his eighth cycle of Axitinib. All treatments were tolerated well without any significant toxicity. Systemic therapies were switched due to disease progression.

**Figure 2:**
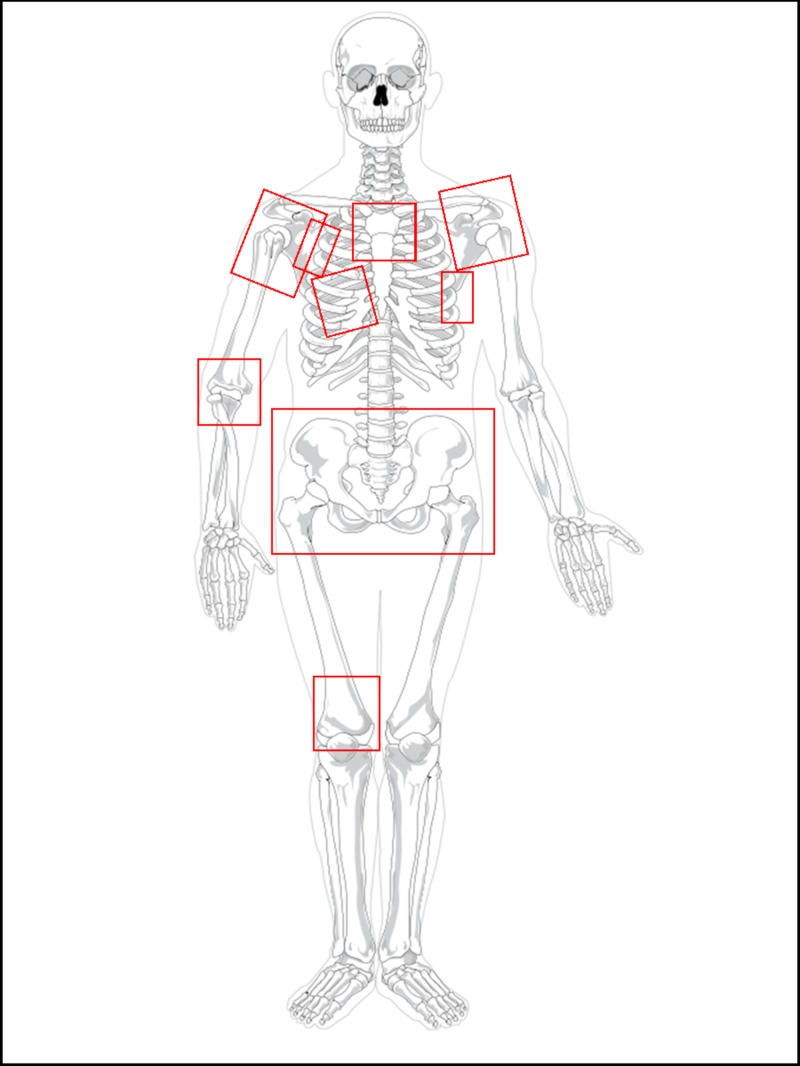
Palliative radiotherapy courses Radiotherapy field has been delineated to demonstrate areas that have been treated throughout the clinical course of the patient.

He has received multiple courses of palliative radiotherapy to manage symptomatic bony metastases. Areas of treatment are illustrated in Figure 2.

**Figure 3:**
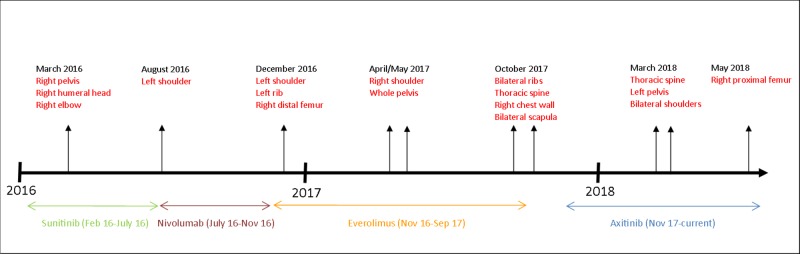
Patient clinical timeline Clinical course of patient is depicted with radiotherapy courses and targets and systemic therapy schedules indicated.

Indications for radiotherapy were largely for poorly controlled pain. He did require radiotherapy for disease at the site of his previous laminectomy that caused recurrent spinal cord compression in March 2018. Because he was educated on the symptoms of cord compression previously, he presented promptly after symptom onset for management. No significant toxicity was observed with treatments. The patient continues to be followed up to this day, and is currently doing well with a palliative performance score (PPS) of 80-90% despite his disease burden and treatment history. His treatment course is illustrated in Figure 3.

## Discussion

The management of mRCC is complex and requires a multidisciplinary approach, often requiring both local and systemic therapies.

Systemic therapy remains the cornerstone of mRCC management. Therapeutic advances in the past decade have played a central role in the clinical improvements we have seen in patients with mRCC. Traditionally, mRCC has been determined to be a chemotherapy resistant disease [5]. Immunotherapies with interferon alpha and interleukin-2 have been the mainstay treatment for mRCC up until this decade. However, response rates were low, with significant accompanying toxicities [1, 6].

With the advent of targeted molecular therapies as well as immune checkpoint inhibitors, the modern armamentarium against mRCC is extensive. Current first line therapy for mRCC in Canada includes the tyrosine kinase inhibitors (TKI) sunitinib and pazopanib. A phase 3 randomized trial showed a significant improvement in progression-free survival (PFS) in patients receiving Sunitinib compared to interferon alpha (HR = 0.42, p < 0.001) [7]. The COMPARZ trial proved that pazopanib was non-inferior to sunitinib in regards to PFS and overall survival (OS), although the toxicity profile differed between the two arms [8]. Temsirolimus, an mTOR inhibitor, has been shown to be superior to interferon-alpha in terms of PFS in patients with poor prognostic factors, and is often used first line in these patients (HR = 0.73, p = 0.008). Prognostic factors included time to metastatic disease, number of metastases, PPS score, hemoglobin, lactate dehydrogenase (LDH), and calcium levels [9].

Systemic therapy options in previously treated patients include mammalian target of rapamycin (mTOR), vascular endothelial growth factor (VEGF), and immune checkpoint inhibitors. Both nivolumab and cabozatinib have shown improved OS when compared with everolimus, and therefore supports their role as second line agents in TKI refractory patients [10, 11]. Immune checkpoint inhibitors in particular, show significant promise in the treatment of mRCC. Most recently, a phase 3 trial comparing nivolumab plus ipilimumab versus sunitinib showed significantly higher overall survival at 18 months in intermediate and poor risk patients, at 75% and 60%, respectively [12].

Surgical resection of oligometastatic lesions has been studied in the non-randomized setting, with results suggesting that the complete resection of metastatic disease may confer a survival benefit. Metastasis site is also of importance, with liver and brain metastases indicative of more widespread disease and worse prognosis [4, 13]. Kavolius et al. identified a number of positive prognostic factors for metastasectomy in recurrent RCC patients, including disease-free interval greater than one year, solitary metastasis and age under 60 years [13].

The low radiation doses and relative paucity of acute toxicity make palliative radiotherapy an important modality as illustrated in our case. A prospective trial showed that in patients treated with 30 Gy, 83% experienced site specific pain relief [14]. The recent advent of stereotactic body radiotherapy (SBRT) has sparked interest in the utility of radiotherapy for local in mRCC. A number of cohort studies have shown greater than 75% rates of local control. A recent systematic review and meta-analysis showed median survival ranged from 11.7 to 22 months, with a low incidence of significant toxicity under 4% [15].

## Conclusions

The treatment paradigm for mRCC has shifted from supportive care to active oncologic management that includes the ongoing role of radiotherapy, systemic therapy, and surgery for controlling symptoms and cancer progression. Our case not only highlights the effectiveness of multidisciplinary management, but also states that patient education about symptoms is important to provide timely care and maintain quality of life. Through our case, we encourage early involvement and close follow-up with radiation and palliative care teams in the management of patients with this complex disease.
